# Super Digit: More Than a Complicated Syndactyly

**DOI:** 10.7759/cureus.28678

**Published:** 2022-09-01

**Authors:** Mariana Agostinho, Vera Eiró, Tatiana Gomes, João Nunes da Costa, Júlio Matias

**Affiliations:** 1 Plastic and Reconstructive Surgery, Centro Hospitalar Lisboa Ocidental, Lisbon, PRT

**Keywords:** wood's classification, hand malformation, syndactyly, congenital, super digit

## Abstract

Super digits are a rare hand malformation, first described by Virchel Wood. Surgical intervention to try to make two fingers out of a super digit has been discouraged. Here, we present a variant of a super digit type IC2 and propose a revision of the characteristics in each super digit subtype. In our view, this adjustment in Wood’s original description could facilitate the identification of super digits, which are a contraindication to syndactyly release.

## Introduction

Super digits are a rare hand malformation, first described by Virchel Wood [1]. Since then, few reports have been published about this, difficult to classify, anomaly. He established two main categories: type I, composed of two metacarpals supporting an oversized finger, and type II, where a single metacarpal supports two or more digits. Super digits type I were even subdivided into: type IA, when two metacarpals support a single, well-defined but enlarged digit; type IB, when there is an incomplete fusion along the longitudinal axis; and type IC, when there is a proximal enlarged delta phalanx articulated either with a normal distal digit (IC1) or two distal digits (IC2). In type II super digits, a single metacarpal can support two normal distal digits (IIA), or a large (IIB1) or split (IIB2) proximal phalanx articulated with two distal digits. Wood’s definition of type IIC and IID is less clear. According to Nunes da Costa and Matias’ interpretation, in type IIC there is a single proximal delta phalanx supporting two almost completely fused distal fingers (IIC1) or two proximal delta phalanges with two fingers with soft-tissue syndactyly (IIC2) [2]. Type IID is associated with polysyndactyly with a single (IID2) or split/multiple proximal phalanges (Type IID1). These authors, based on a single case, proposed a new category (type III) where two divergent metacarpal bones and two well-defined proximal phalanges, fused only at their distal part, support a distal digit. Here, we report a clinical case of a child with complicated syndactyly on the left hand that in our view, could be considered a super digit type IC2.

## Case presentation

**Figure 1:**
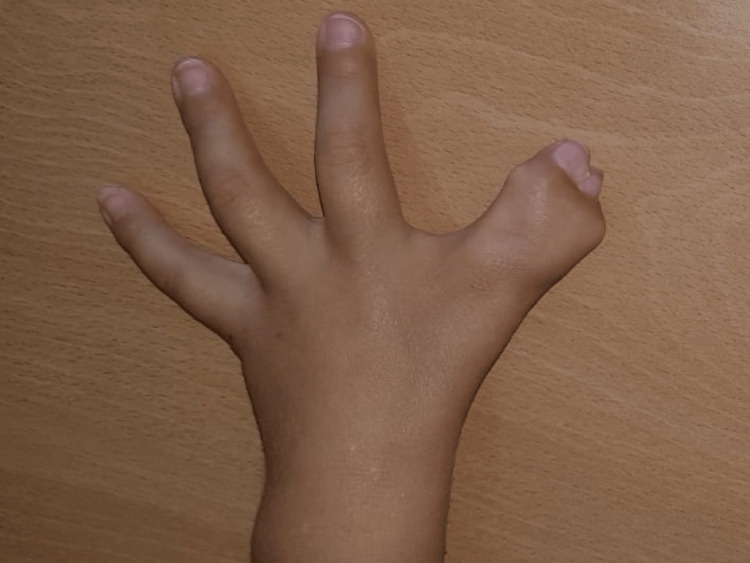
The appearance of the left hand

**Figure 2:**
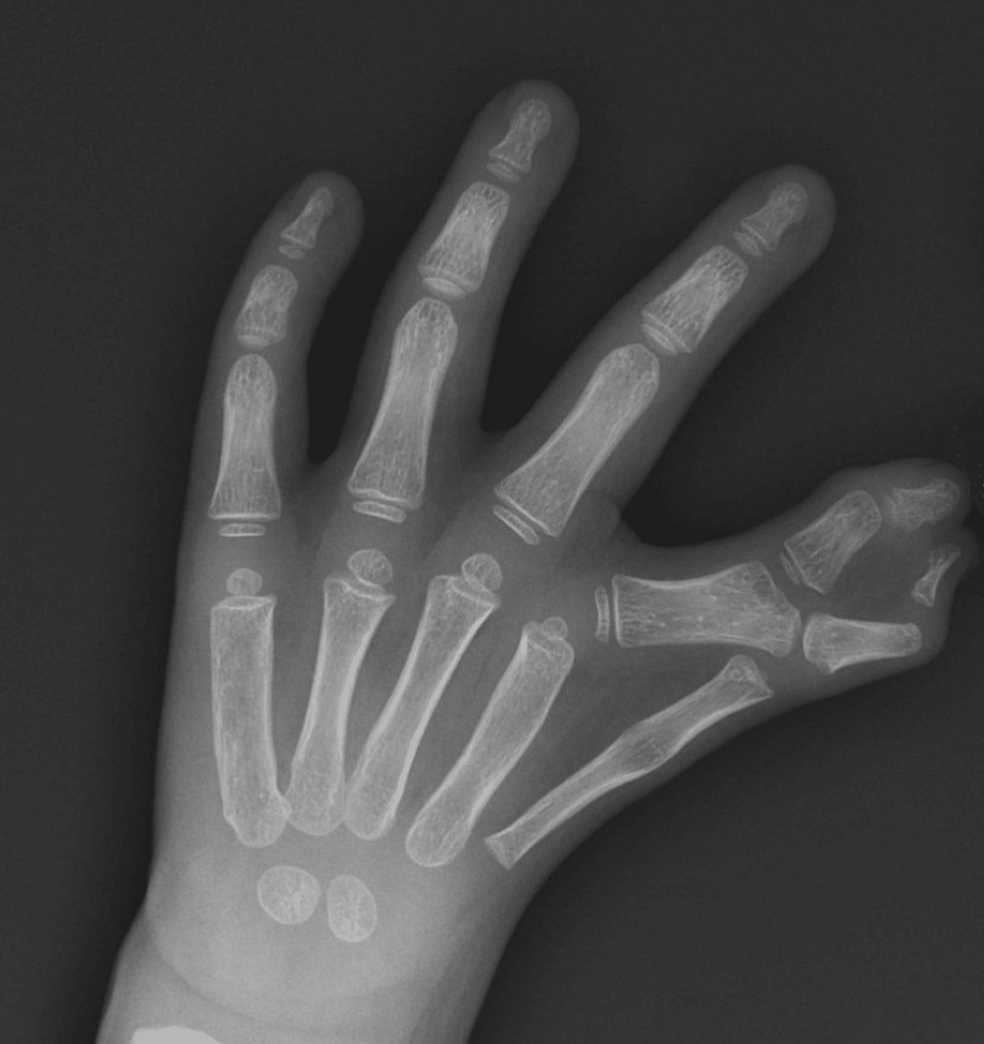
Radiograph of the left hand

A four-year-old boy, with otherwise normal development, presented bilateral hand anomalies. Radial hand malformations were limiting pinch and grasp. Radiographs showed a five-fingered hand on the right, and complicated syndactyly of the first interdigital space, on the left. In this report, we will focus on the left hand (Figure 1) to describe what we believe to be more than a side-to-side fusion of digits. From radiographs (Figure 2) and noticing the first and second metacarpals, we observed that the more radial one had an elongated and narrow conformation, probably the result of fusion of the metacarpal to a phalanx. These were articulated with a common proximal phalanx with an enlarged and pentagonal shape, supporting two middle and two distal phalanges with a divergent-convergent configuration.

**Figure 3:**
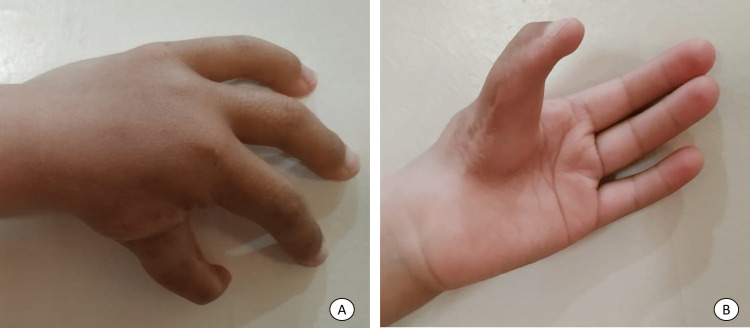
Left hand 18 months after surgery A: Dorsal side; B: Volar side

The patient underwent a simultaneous, bilateral pollicization, with the first ray ablation on the left hand. At 18 months of follow-up, he has a stable and mobile thumb bilaterally without angulation and is acquiring grasp and dexterity capacity (Figure 3).

## Discussion

Super digits represent a rare clinical entity often associated with several anomalies and syndromes. Two main types have been formally described as explained above. From his series, Wood concluded that loss of motion, angulation, undergrowth, and nonfunction are the most common outcomes of a non-corrected super digit. He discouraged surgery to try to make two fingers out of a super digit. Dao et al. were of the same opinion [3]. They added that consideration must be given to whether one finger should be sacrificed to produce a more functional three or four-fingered hand.

Regarding our case, we consider that the anomaly present is a variant of Wood’s super digit type IC2 [1]. According to Wood’s classification, type IC2 consisted of two metacarpals holding a delta-proximal phalanx that supports two distal digits. At that time, he only identified one representative case. Our patient’s features do not totally match the original description of a super digit type IC2. For this reason, we would suggest that this category could be expanded to include all forms of atypical and large proximal phalanges if more similar cases were found. Likewise, it could be interesting to span the characteristics of each super digit subtype. These adjustments in Wood’s original description, in combination with clarification of subtypes IIC and IID, and maybe the addition of a third subtype, as already proposed, could help to recognize more super digits, where planning a syndactyly release will not bring the best results.

## Conclusions

Super digits are a rare clinical entity. The term was developed by Wood and since then, the concept has seen little development. With this case report, the authors aimed to revisit Wood’s classification of the super digit. The authors suggest an expansion of super digit type IC2 characteristics to include all forms of atypical and large proximal phalanges and present a clinical example.

These adjustments in Wood’s original description could help to recognize more super digits and improve the development of a treatment plan.
